# Optimization of Bio-Foamed Concrete Brick Strength via Bacteria Based Self-Healing and Bio-Sequestration of CO_2_

**DOI:** 10.3390/ma14164575

**Published:** 2021-08-14

**Authors:** Abdullah Faisal Alshalif, J. M. Irwan, Husnul Azan Tajarudin, N. Othman, A. A. Al-Gheethi, S. Shamsudin, Wahid Ali Hamood Altowayti, Saddam Abo Sabah

**Affiliations:** 1 Jamilus Research Centre for Sustainable Construction (JRC-SC), Faculty of Civil Engineering and Built Environment, Universiti Tun Hussein Onn Malaysia, Parit Raja 86400, Johor, Malaysia; saddam@uthm.edu.my; 2 Division of Bioprocess, School of Industrial Technology, Universiti Sains Malaysia, Gelugor 11800, Pulau Pinang, Malaysia; 3 Micro-Pollutant Research Centre (MPRC), Faculty of Civil Engineering and Built Environment, Universiti Tun Hussein Onn Malaysia, Parit Raja 86400, Johor, Malaysia; norzila@uthm.edu.my (N.O.); adel@uthm.edu.my (A.A.A.-G.); wahidali@uthm.edu.my (W.A.H.A.); 4 Sustainable Manufacturing and Recycling Technology, Advanced Manufacturing and Materials Center (SMART-AMMC), Universiti Tun Hussein Onn Malaysia, Parit Raja 86400, Johor, Malaysia; shazarel@uthm.edu.my

**Keywords:** self-healing, CO_2_ capture, CaCO_3_ precipitation, *Bacillus tequilensis*, carbonic anhydrase, urease

## Abstract

This research aimed to optimize the compressive strength of bio-foamed concrete brick (B-FCB) via a combination of the natural sequestration of CO_2_ and the bio-reaction of *B. tequilensis* enzymes. The experiments were guided by two optimization methods, namely, 2^k^ factorial and response surface methodology (RSM). The 2^k^ factorial analysis was carried out to screen the important factors; then, RSM analysis was performed to optimize the compressive strength of B-FCB. Four factors, namely, density (D), *B. tequilensis* concentration (B), temperature (T), and CO_2_ concentration, were selectively varied during the study. The optimum compressive strength of B-FCB was 8.22 MPa, as deduced from the following conditions: 10% CO_2_, 3 × 10^7^ cell/mL of B, 27 °C of T and 1800 kg/m^3^ of D after 28 days. The use of *B. tequilensis* in B-FCB improved the compressive strength by 35.5% compared to the foamed concrete brick (FCB) after 28 days. A microstructure analysis by scanning electronic microscopy (SEM), energy dispersive X-ray (EDX) and X-ray diffraction analysis (XRD) reflected the changes in chemical element levels and calcium carbonate (CaCO_3_) precipitation in the B-FCB pores. This was due to the *B. tequilensis* surface reactions of carbonic anhydrase (CA) and urease enzyme with calcium in cement and sequestered CO_2_ during the curing time.

## 1. Introduction

Global carbon dioxide (CO_2_) emissions have increased in recent decades, in line with increases in anthropogenic activities. Therefore, a great deal of research has been conducted to reduce the impacts of the catastrophic environmental issues due to CO_2_ emission such as global warming, rising sea levels, and climate change [1]. Fossil fuel combustion and cement manufacturing are the biggest contributors to CO_2_ emissions, representing around 88% [2]. The process of producing one ton of cement emits around 900 to 1000 kg of CO_2_ due to the energy required to burn limestone [3,4]. Between 2005 and 2015, cement production increased worldwide by 79.5%, i.e., from 2284 to 4100 Mt/yr [2,4]. For this reason, various studies on concrete technology have focused on reducing cement production by using alternative materials [5,6]. However, the demand and production of cement continue to increase, resulting in increased global emissions of CO_2_. Furthermore, most of the replacement materials used in concrete reduce its strength.

Due to the ability of bacteria to improve the physical and mechanical properties, particularly compressive strength, of concrete, so-called bio-concrete has become popular worldwide [7,8]. Many researchers have realized the potential of bacteria to increase the strength of bio-concrete through a self-healing process. Bacteria are typically used in different types of concrete such as normal, fly ash, and rice husk ash. Different types of bacteria have been used for this purpose, such as *Bacillus pasteurii*, *Pseudomonas aeruginosa*, *B. alkalinitrilicus*, *B. sphaericus*, *B. subtilis*, *Enterococcus faecalis*, *Shewanella* sp., *S. pasteurii* and *Ureolytic* [7]. However, the use of bacteria in foamed concrete has not been reported in previous studies, according to the latest update of the Scopus database in 2019. Bacteria have the potential to be more effective in foamed concrete than in normal concrete due to the high level of porosity and availability of oxygen formed by the foaming agent in the former [9].

Challenges to the use of bacteria in bio-concrete, such as overcoming the highly alkaline and anaerobic conditions [10], have given rise to the use of silica gel, capsules, and adaptation media [11]. Furthermore, the ability of bacteria to produce enzymes such as urease or carbonic anhydrase (CA) is critical to the precipitation of calcium carbonate (CaCO_3_) on the surface, which results in self-healing of the concrete pores. The healing of bio-concrete pores occurs due to the reaction of urease or CA enzymes with the available cement-based calcium ions (Ca^+^) on the surface of the bacteria. Typically, the reaction results in the precipitation of CaCO_3_, which improves the compressive strength [12,13]. The reaction of urease and CA enzymes enhances the natural carbonation reaction of bio-concrete with bicarbonate (CO_3_^2−^), which further reacts with the calcium in cement to increase CaCO_3_ precipitation and accelerates carbonation [14,15].

According to Rafat Siddique (2011), bacteria concentration is one of the most critical factors affecting the performance and compressive strength of bio-concrete [16]. While increasing the bacteria concentration increases compressive strength, exceeding the optimum value can have a negative effect [17]. Furthermore, higher cell concentrations disrupt the integrity of the matrix due to excessive microbial activity. The various factors that affect the compressive strength of bio-concrete may be categorized the solid physical properties, material chemical properties, and external conditions of the environment [18]. Therefore, the effect of bacteria and other factors must be investigated to optimize the compressive strength of bio-concrete.

This research aims to use accelerated CO_2_ production via *Bacillus tequilensis* (*B. tequilensis*) to optimize the strength of bio-foamed concrete brick B-FCB, using a 2^k^ factorial design and response surface methodology (RSM) using the Minitab 18 software to complete the analysis. *B. tequilensis* concentration (B), the density of B-FCB (D), CO_2_ concentration (CO_2_) and temperature (T) of curing in the chamber were carefully investigated before optimization of the compressive strength of B-FCB.

## 2. Materials and Methods

In this study, the materials used to prepare the foamed concrete were cement, sand, water and a foam agent, while *B. tequilensis* was added to produce a new type of concrete, namely, bio-foamed concrete. The two methods used to optimize the compressive strength of B-FCB were 2^k^ factorial design and RSM. Details regarding the materials and methods used in this study are provided in the following subsections.

### 2.1. Materials

#### 2.1.1. Cement

Ordinary Portland cement (OPC) manufactured by Cement Industries of Malaysia Berhad (CIMA), type I, MS 522 according to American society for testing and materials (ASTM), was used in this study. The composition and specifications of the OPC are defined in BS 197-1:2000, as shown in Table 1. The quantity of cement was adjusted according to the density used in the foamed concrete mixture with and without *B. tequilensis.*

#### 2.1.2. Sand

**Figure 1:**
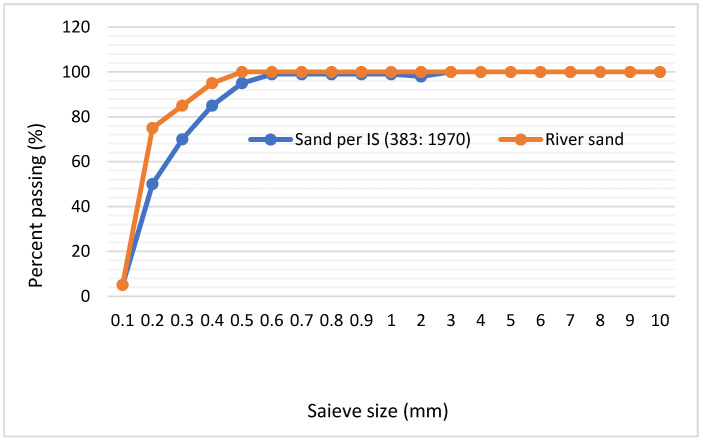
Grading curve for river sand.

River sand was sieved using a plate passing 1 mm, according to IS 383:1970 [19,20]. The particle size distribution is presented in Figure 1. The sieved sand was placed in an oven at 100 °C for 24 h to dry and to remove any microorganisms present. It was then cooled at room temperature before use in the concrete mixture.

#### 2.1.3. Water

Water was used with a foamed concrete mix to produce the FCB. The same water was used with *B. tequilensis* added in powdered form to produce B-FCB; the water containing *B. tequilensis* was agitated to ensure that the powder was evenly distributed.

#### 2.1.4. Bacteria

The *B. tequilensis* was isolated from five samples of cement kiln dust (CKD), which has an extreme pH value and provides anaerobic conditions. The isolation of *B. tequilensis* was subjected to several tests, namely, CA and urease assays, growth in theioglycollate, a candle jar test and growth in a bio-foamed concrete medium [21]. Then, the most resilient bacteria were isolated from the five samples, and tested for the following properties: ability to produce CA and urease enzymes, facultative anaerobic, and capable of growth in high concentration of CO_2_ and in a bio-foamed concrete simulation medium. After that, a powdered form of *B. tequilensis* was produced to control its concentration in the concrete mixture: a bacteria pellet was placed into a freeze dryer with the following settings: −40 °C and 0.133 mbar of pressure for 96 h [21].

In this research *B. tequilensis* was selected as one of the factors used to optimize the compressive strength of B-FCB. The concentration of *B. tequilensis* was suggested by the 2^k^ factorial and RSM methods shown in Table 2.

#### 2.1.5. Foam Agent

A synthetic type CF 500 foaming agent with a density between 45–65 kg/m^3^ and an expansion ratio of 1:20 was used in this study to produce air bubbles in the foamed concrete mixtures with and without bacteria. The foaming agent was diluted with water at a ratio of 1:20 and aerated to a density of 65 kg/m^3^, according to ASTM C796 [20,22].

### 2.2. Methods

#### 2.2.1. Design of Experiments

In this study, the optimization of the compressive strength of B-FCB involved two stages. The first stage, called screening experiments, was carried out according to the 2^k^ factorial design method, consisting of 11 runs with three center runs added for the curvature test analysis. The 2^k^ factorial design is the most commonly used technique in design of experiments (DOE), and was very helpful in determining the important factors of the experiment. Due to time and funding constraints, the 2^k^ factorial design was prioritized. The experiment was run based on the 2^3^ full factorial design and was performed to study the effects of the four process parameters shown in Table 3. The low- and high-level settings of several factors, particularly the *B. tequilensis* concentration (B), density of the concrete (D), temperature (T) and CO_2_ concentration (CO_2_), were input and analyzed using Minitab software (version 18, Pennsylvania State University, State College, PA, USA) and analyzed. A reasonable range for each factor was selected according to previous research findings [2], as presented in Table 3. The next stage used to optimize the compressive strength of B-FCB by RSM analysis involved adding eight axial and two additional runs at the center points. Hence, a total of 21 experiments were performed, comprising eight factorial runs, eight axial runs, and five center runs. This certainly increased the accuracy levels of the empirical models deduced from the Analysis of Variance (ANOVA) data, as shown in Equation (1).

(1) $$Y=\beta_{0}+\beta_{1}x_{1}+\beta_{2}x_{2}+\beta_{11}x^{2}{}_{1}+\beta_{22}x^{2}{}_{2}+\beta_{12}x_{1}x_{2}$$

where

*Y* is the response (output function)

*x*_1_, *x*_2_, …., *x_k_* are the factors (Input variables)

*β*_0_ is the grand average of all observations

*β*_1_ is half of the *A* effect

*β*_2_ is half of the *B* effect.

ANOVA was also used to rank the main effects and analyze the interactions between the input factors. ANOVA is considered essential for structured analyses of results in the 2^k^ design.

#### 2.2.2. Mixture Design

The mixture design of each run was based on the suggested densities and *B. tequilensis* concentration. The mass of the solid materials (cement/sand) was distributed in the ratio of 1:1.35 according to ACI 523.3R, using the trial method of mix design [20]. Details of the experimental runs are shown in Table 4. The first 11 runs were suggested by the 2^k^ factorial design for the screening stage, whereas the last 10 runs were added to complete the RSM analysis using the Minitab 18 software.

#### 2.2.3. Specimen Preparation and Curing

FCB and B-FCB specimens were prepared with the following dimensions: 215 mm × 100 mm × 75 mm. The total number of specimens for each run was six, comprising three 3 without (as controls) and three with *B. tequilensis*. The specimens were demolded for 24 h before oven drying at 50 °C for 72 h to avoid water evaporation, which has the potential cause chemical reactions with the available CO_2_ in the chamber, resulting in a decrease in the CO_2_ concentration during the process. The FCB and B-FCB specimens were subjected to curing in the chamber under different run conditions (T and CO_2_) for 28 days, as suggested by the 2^k^ factorial and RSM methods, as shown in Table 4.

## 3. Results and Discussion

### 3.1. Screening Stage Analysis (2^k^ Factorial Design)

The results of the compressive strength experiments during screening are presented in Table 5. For one replicate design, the internal error could not be estimated. Therefore, all high-order interactions were neglected and the mean squares of the omitted factors were combined to estimate the error. The percentage of R^2^ was 96.08%, which reflects the highly significant effect of these factors on the compressive strength of B-FCB. This proved that the experimental results could be reproduced with high repeatability and accuracy.

The results of the screening stage presented in Table 5 and Table 6 indicate that all the selected factors significantly influenced the response; as such, they were retained for the RSM analysis. The discussion of the results is divided into two parts to assist in the interpretation of the ANOVA of the DOE, optimization, and modelling of the compressive strength of B-FCB.

### 3.2. Compressive Strength of B-FCB (2^k^ Factorial Analysis)

#### 3.2.1. ANOVA Analysis, Main Effect and Interaction Plots

This section presents the effects of each factor, i.e., B, D, T and CO_2_, on the compressive strength of B-FCB. The compressive strength of each run was analyzed to identify the effects of and interactions among the factors.

The results in Table 6 show the effects of and interactions among the factors on the compressive strength of the B-FCB specimens after 28 days under controlled curing conditions in a chamber. CO_2_, T and D significantly influenced the compressive strength of B-FCB, as evidenced by the low *p*-values (<0.05) 0.012, 0.003 and 0.000, respectively). B was insignificant because its *p* > 0.05; however, a significant interaction with CO_2_ was observed.

**Figure 2:**
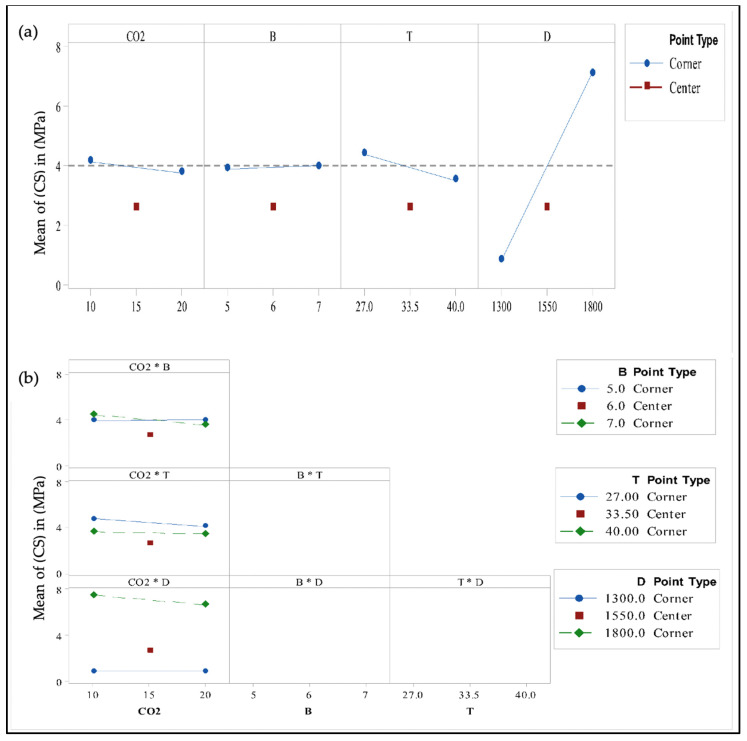
Results of factorial analysis with curvature test: (**a**) Main effect plot of compressive strength, and (**b**) Interaction plot of compressive strength.

The main plot shown in Figure 2a presents the effect of each factor on the compressive strength of B-FCB. The values of the main factors of D, T, CO_2_ and B were 6.2525, 0.8575, 0.3975 and 0.0925, respectively, as presented in Table 6. As observed, factor D had the highest effect on the compressive strength, whereby an increase in D led to greater compressive strength, as presented in Figure 2a. However, the compressive strength of B-FCB was higher with a lower level of T, i.e., an increase in T had a negative effect on the strength of B-FCB. In addition, an increase in CO_2_ concentration during curing in the chamber caused a reduction in compressive strength. According to the ANOVA results, and compared to other factors, *B. tequilensis* alone was less likely to influence the compressive strength.

The interactions between CO_2_*B, CO_2_*T, and CO_2_*D had *p*-values < 0.05, as shown in Table 6 and Figure 2b, which means that all interactions had a significant effect on compressive strength; however, the highest interaction was for CO_2_*B with P, with effect values of 0.008 and 0.4800, respectively. The strength of B-FCB increased with a high level of B and low level of CO_2_, but decreased with a high level of CO_2_. This finding confirmed the strong relationship between B and CO_2_ and the reactions with CA and urease enzymes to accelerate CO_2_ sequestration in the B-FCB pores to form CaCO_3_. The cumulative formation of CaCO_3_ led to the high compressive strength of B-FCB. In contrast, the increase or decrease of CO_2_ with a low level of B did not have a strong effect on compressive strength. The strength of B-FCB improved with a low level of T and CO_2_, while it decreased with higher levels of T and CO_2_. The CO_2_*T interaction can be interpreted as follows: a high level of CO_2_ during the curing period may restrict the activities of bacteria enzymes, resulting in reduced CaCO_3_ formation. This finding is supported by the steep line of CO_2_ performance shown in Figure 2a. The interaction between CO_2_*D shows a drastic change in the compressive strength of B-FCB when D is at a high level and CO_2_ at a low level. However, at the same level of D, an increase in CO_2_ to 20% lowered the compressive strength. This result indicated that by increasing the CO_2_ to 20%, the restriction of enzymatic reactions suppressed the ability of the bacteria to generate CaCO_3_ on the surface and heal the pores of B-FCB.

The ANOVA results confirmed that the factors and interactions had a significant effect on the compressive strength of B-FCB. A ranking of each factor is presented in Table 6.

#### 3.2.2. Cube Plot of B-FCB Compressive Strength

**Figure 3:**
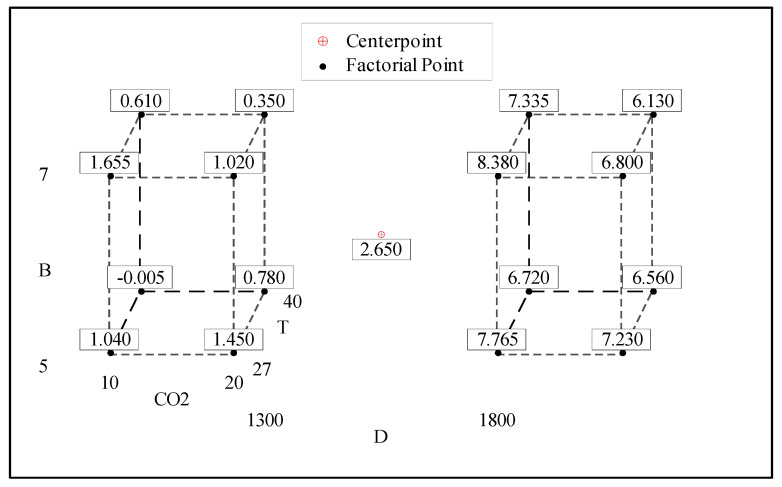
Cube plot of compressive strength.

The relationship between the factors and response (compressive strength) can be analyzed from the cube plots shown in Figure 3. The predicted values for each combination of factor levels, split by different densities of 1300 kg/m^3^ and 1800 kg/m^3^ D, are depicted in the corners of the plots. The maximum compressive strength was 8.38 MPa, which appeared with a high level of D (1800 kg/m^3^) and B when T and CO_2_ were set at low levels. The compressive strength with a low level of D (1300 kg/m^3^), CO_2_ and T was 1.74 when B remained at a high level. It can therefore be concluded that in order to obtain the highest compressive strength, factor B must be set to a high level, while the CO_2_ and T should remain low, regardless of the density setting. By applying these parameters, favorable conditions may be established for *B. tequilensis* to become more reactive to CA and urease enzymes; this will accelerate the sequestration of CO_2_ from the atmosphere and greatly improve the healing process of B-FCB via the formation of abundant CaCO_3_.

The increase in compressive strength due to an increase in B has already been confirmed in previous research works [23,24]. Typically, an increase of bacteria resulted in an increase in CaCO_3_ formation on the surface that healed the pores in the concrete [23]. Therefore, it can be inferred that the healing process in bio-concrete pores leads to an increase in compressive strength. However, higher bacteria concentrations occasionally led to excessive bacteria activity, which disrupted the matrix integrity and ultimately decreased the compressive strength [17].

### 3.3. Analysis of Response Surface Methodology (RSM) Results

The 2^k^ factorial analysis proved that all the factors in this study had significant effects on the responses, as discussed in Section 3.2. RSM analysis was applied to optimize the compressive strength of B-FCB after the screening stage. The effect and correlation of the four selected factors were further analyzed using RSM. The Minitab 18 software suggested that 10 more runs be added to the previous eleven runs shown in Table 7, according to central composite design (CCD). Two out of ten runs were assigned to center points, and therefore, the full RSM design comprised five center points. The optimization results for compressive strength are discussed in detail in the following sections.

#### 3.3.1. Optimisation of Compressive Strength for B-FCB

RSM was used to optimize the compressive strength of B-FCB using residual plot, ANOVA analyses, along with the surface, contour, and optimization plots, as discussed in detail in the following sections.

##### Residual Plot for B-FCB Compressive Strength

**Figure 4:**
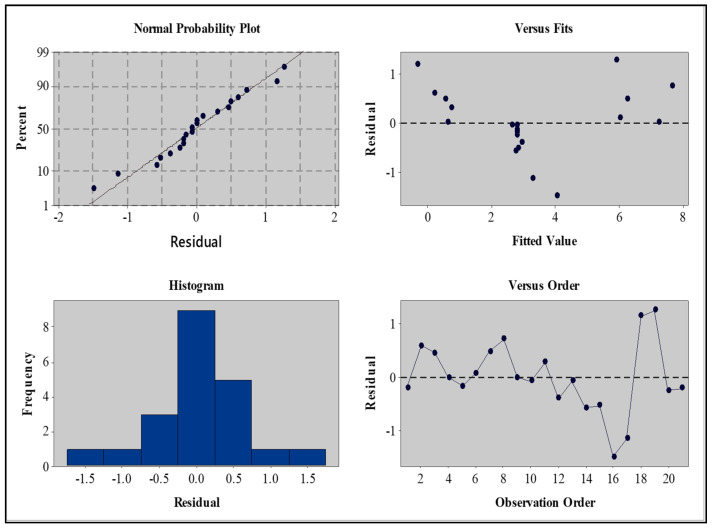
Residual plot for compressive strength of B-FCB.

The residual plots of the DOE analysis helped us to evaluate the accuracy of the data. The effects of the nuisance factors were not included in the analysis, except when error measurements were performed. The data distribution must be normal and independent from the zero mean value and constant variance to ensure that the *F_o_* ratio follows the (F) distribution [25]. The distribution of the compressive strength data of B-FCB is present in the residual plots of the normal probability shown in Figure 4.

The presented data indicate that the model meets the assumptions of the analysis. Furthermore, most points follow a straight line, with only three drifting away from the central data. In addition, the residual versus fitted plots presented randomly dispersed data around zero with no clear observable pattern. However, three points exceeded the limit, which confirmed that the error is normal in the results regarding compressive strength. The permissible error to conclude the findings was below 10%, which indicated a high level of accuracy in the data analysis. The fine segregation of the points around the normal probability line demonstrated a precise prediction of the B-FCB strength.

##### Analysis of RSM-ANOVA for Compressive Strength of B-FCB

The ANOVA results of compressive strength were analyzed using RSM once all the insignificant factors had been removed, as presented in Table 8.

D, T, and CO_2_ were found to be the most significant factors in the analysis, as indicated by *p* < 0.05, whereas the B was insignificant due to *p* > 0.05. Furthermore, the square of density (D*D) also showed a highly significant effect, which confirmed that a strong relationship exists between density and compressive strength in B-FCB. In contrast, interactions CO_2_*B and CO_2_*D were shown to be highly significant, i.e., *p* < 0.05. As observed, the effect of factor B alone was insignificant. However, the CO_2_*B interaction demonstrated a highly significant effect with a *p*-value equal to 0.003, as shown in Table 8. This finding corroborates the theory that the CA and urease enzymes secreted by the bacteria chemically reacted with atmospheric CO_2_, resulting in the formation of CaCO_3_ on the surface [26,27]. Therefore, it can be inferred that the CO_2_*B interaction considerably affected the compressive strength by healing the pores of B-FCB by CaCO_3_ precipitation [28].

##### Response Surface and Contour Plots for the Compressive Strength of B-FCB

**Figure 5:**
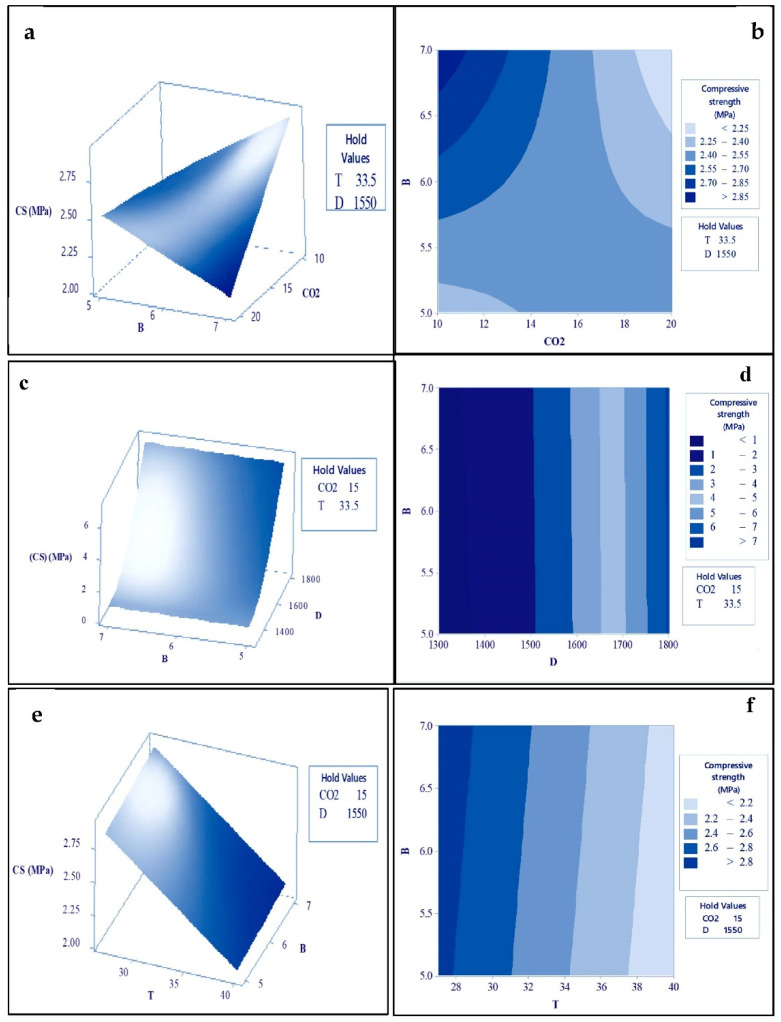
Response surface and contour plots for compressive strength of B-FCB; (**a**,**b**) between B and CO_2_, (**c**,**d**) between B and D and (**e**,**f**) between B and T.

The response surface plots shown in Figure 5a,c,e depict the effects of the various parameters on the compressive strength of B-FCB. The contour plots played a significant role in the creation of the response surface analysis, which includes layers with different graded colors indicating possible independence of factors to a response, as depicted in Figure 5b,d,f. Both the surface and contour plots graphically depict the relationship between each two process factors and compressive strength, while the other two associated factors are maintained at the center value.

Figure 5a,b depict the effect of B and CO_2_ concentrations on the compressive strength of B-FCB. As observed, the compressive strength was maximized with a high level of B and a low level of CO_2_. In contrast, the compressive strength of B-FCB was lower with higher levels of B and CO_2_. Based on these findings, it can be concluded that B favors lower levels of CO_2_ during curing to accelerate the sequestration of CO_2_, leading to the precipitation of more CaCO_3_.

The effect of B and D did not show a strong relationship, as depicted in Figure 5c,d. Typically, D is the main factor that controls the strength of the foamed concrete, and both were found to exhibit a linear relationship [20,21,22,29,30]. However, the weakness in the relationship between B and D was attributed to an insufficient curing period that strongly affected the level of compressive strength of B-FCB. In addition, due to the high level of porosity in B-FCB compared to other types of concretes, the 28-day period for curing was insufficient to heal the pores. Furthermore, it was observed that when the D was set at higher levels, the resulting compressive strength was also higher at all levels of B. This was due to the fact that at higher levels of D, the bricks were highly porous and could easily be healed within 28 days.

The influence of B and T on compressive strength is shown in Figure 5e,f. The optimal compressive strength occurred when T was retained between 27 °C and 29 °C, while a lower compressive strength was observed with T ranging from 38 °C to 40 °C. Therefore, an increase in B with a low level of T will marginally improve the compressive strength. However, the compressive strength is greater when B is set at a low level and T at a high level. According to the previous observations, it can be concluded that the improvement of compressive strength in B-FCB cannot be attributed to a single factor; rather, interactions of B with CO_2_, D, and T must be carefully evaluated to optimize the compressive strength.

##### Optimization Plots for Compressive Strength of B-FCB

**Figure 6:**
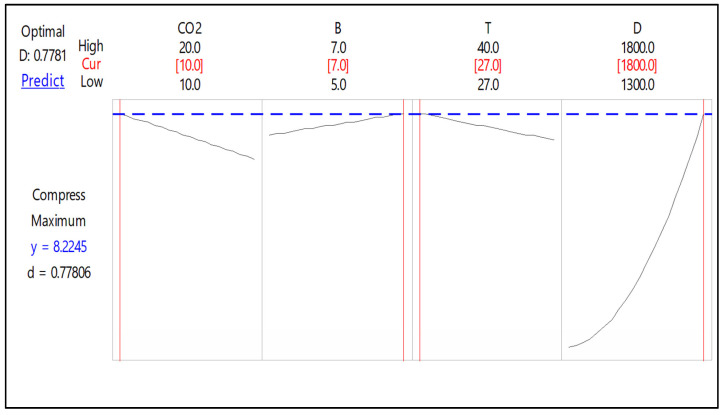
Optimization plot for the compressive strength of B-FCB.

The optimization plot shows how different experimental settings affected the predicted compressive strength of B-FCB. Figure 6 shows that the single desirability (*d*) and response (*y*) were 0.77806 and 8.2245, respectively. The red solid lines indicated the values of each factor that led to the highest compressive strength. Meanwhile, the dotted blue lines represent the predicted compressive strengths.

A decrease in CO_2_ and T during curing process increased the compressive strength of B-FCB, while the opposite result was observed with higher levels of B and D. The resulting predicted values of D and T were in accordance with data from a previous study, whereby the compressive strength improved when D was set at a high level but T was below 30 °C [31]. The highest predicted response for compressive strength was obtained under the following conditions: CO_2_, B, T and D were set at 10%, 30 × 10^7^ cell/mL, 27 °C, and 1800 kg/m^3^, respectively.

An empirical model was developed via RSM analysis after optimizing the compressive strength of B-FCB. The model derived from the ANOVA results indicated a clear relationship between the independent variables (significant terms) and compressive strength response. The final regression equation in uncoded units of compressive strength is given in Equation (2).

Compressive strength (MPa) = 34.05 + 0.575 CO_2_ + 0.836 B − 0.05923 T − 0.05942 D + 0.000024 D*D − 0.0522 CO_2_*B − 0.000189 CO_2_*D (2)

#### 3.3.2. Comparison of Compressive Strength between FCB and B-FCB

**Figure 7:**
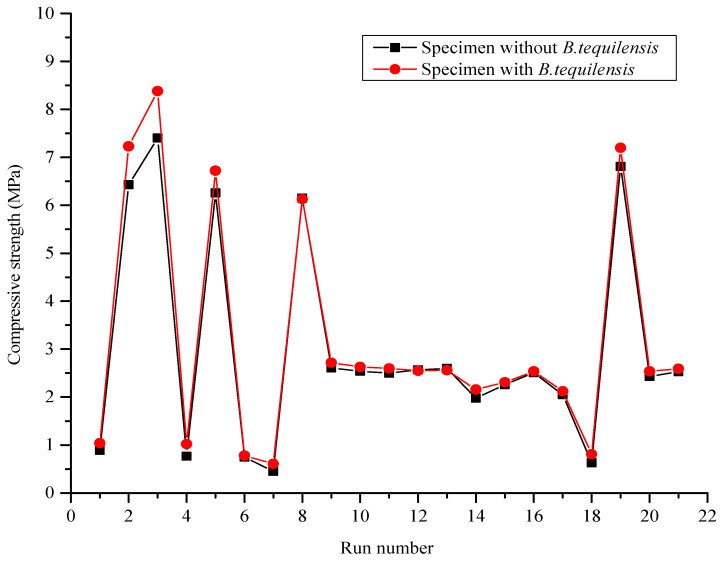
Compressive strength of FCB and B-FCB.

Compressive strength was considered the key variable in this study of B-FCB concrete. The results indicated that D was the main factor influencing the increase or decrease in compressive strength in concrete specimens with or without *B. tequilensis*. The results in Figure 7 present the differences in the compressive strengths between FCB and B-FCB. The mixtures with run numbers 2, 3, 5, 8, and 19 at D = 1800 kg/m^3^ displayed the highest compressive strength of the 21 runs, while the compressive strength of B-FCB was enhanced by 12.4%, 13.2%, 7.3%, −0.3%, 2.0%, respectively, compared to the compressive strength of FCB. However, the highest compressive strength from among the 21 runs for both FCB and B-FCB was run 3, with the compressive strength values of 7.40 MPa and 8.38 MPa, respectively.

The performance of the mixtures with D = 1300 kg/m^3^ was similar in terms of compressive strength to runs at 1800 kg/m^3^. However, the improvement in the compressive strength of B-FCB in runs 1, 4, 6, 7, and 18 was higher than that of FCB by 17.5%, 32.5%, 4.0%, 35.5% and 11.8%, respectively. In addition, runs at D = 1550 kg/m^3^ yielded similar results. However, D was not the only factor responsible for changes in the compressive strength results for FCB and B-FCB. Other factors, namely, T, CO_2_, and B, also showed an influence on the compressive strength performance.

Furthermore, runs 16 and 17 clearly revealed the effect of T on the compressive strength of FCB and B-FCB concrete. Other conditions in runs 16 and 17 were kept constant, while the degree of T changed from 27 °C to 40 °C. It was observed that the compressive strength for both FCB and B-FCB decreased from 2.51 MPa to 2.05 MPa and 2.54 MPa to 2.12 MPa for the specimens, respectively. Additionally, there was an improvement in the compressive strength for similar runs of B-FCB at 1.2% and 3.3%, compared to FCB, respectively.

The previous statement illustrates that B-FCB has a higher compressive strength compared to FCB at different D levels. In fact, the higher compressive strength was demonstrated by runs with higher levels of D. However, the improvements between FCB and B-FCB were achieved in runs using lower levels of D. Typically, *B. tequilensis* plays a vital role in improving the compressive strength of B-FCB by precipitating high quantities of CaCO_3_, which heals pores on its surface. Thus, the pore-healing process was more evident in specimens with low D of B-FCB compared to those with high D. This was because the specimens with low D were characterized by high porosity compared to those with high D.

These findings reveal the trend of compressive strength results, and confirm that compressive strength is affected by numerous factors. Thus, considering only one factor may yield defective FCB and B-FCB. Consequently, in this study, 21 runs were used in RSM to analyze and optimize the compressive strength of B-FCB according to four main variables and their interactions.

## 4. Microstructure Analysis

A microstructure analysis was conducted by SEM, EDX, and XRD to investigate the healing of specimen pores, weight changes in chemical elements, and comparative precipitation of CaCO_3_ in B-FCB and FCB.

### 4.1. Healing Process and Porosity Determination

**Figure 8:**
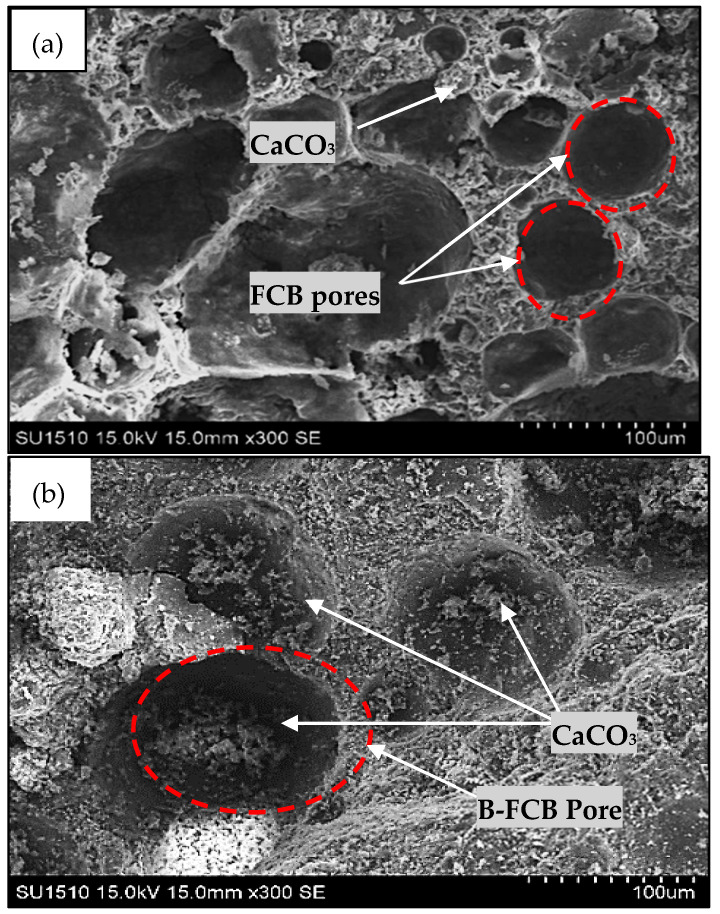
SEM images with specimens with 1300 kg/m^3^ density of (**a**) FCB and (**b**) B-FCB at 28 days.

SEM images were used to demonstrate the healing process and determine the porosity of the FCB and B-FCB specimens, as presented in Figure 8. In general, the healing process of B-FCB pores was clearly present, compared to the FCB pores. The healing process of the pores was due to the formation CaCO_3_ on the surface in the B-FCB pores. This occurs due to the chemical reaction between the sequestrated CO_2_ and the CA and urease enzymes of *B. tequilensis* during the curing process.

However, some pores did not fully heal, either due to the high level of porosity in the specimens or inadequate curing time to precipitate more CaCO_3_ in the pores, as shown in Figure 8. This implies that the effectiveness of the high level of CO_2_ sequestrated in B-FCB is responsible for the increase in CaCO_3_ precipitation that accelerates the healing process. In addition, the relationship between the healing of the B-FCB pores and the high level of CO_2_ during curing confirms the ability of the bacteria to accelerate the CO_2_ sequestration process. This can be considered a future direction for CO_2_ sequestration technology [2]. The results also confirmed that the high compressive strength performance of B-FCB compared to FCB was due to the healing of B-FCB pores. The inference is that the high performance of the compressive strength observed in B-FCB compared to FCB was due to the healing of B-FCB pores by precipitation of CaCO_3_. Conversely, the availability of CA and urease enzymes in the *B. tequilensis* used in B-FCB helped accelerate CO_2_ sequestration and CaCO_3_ formation on the *B. tequilensis* surface, which gave rise to the healing process observed in Figure 8 [32].

### 4.2. Elemental Analysis of FCB and B-FCB

**Figure 9:**
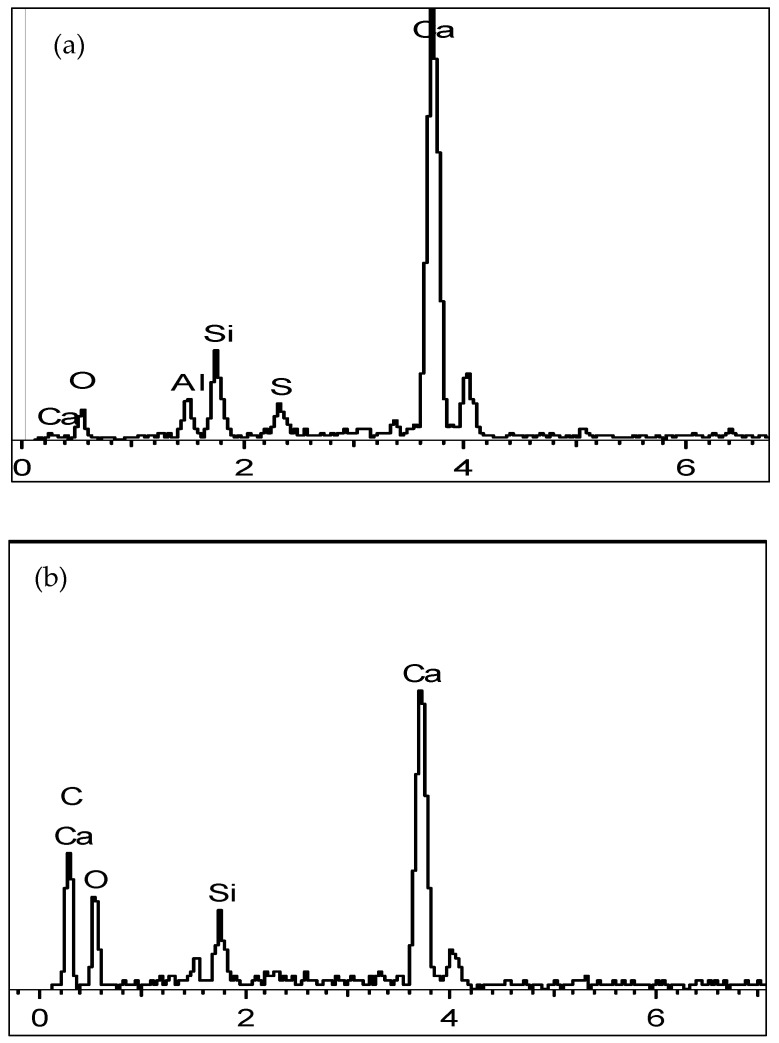
Comparative EDX analyses between (**a**) FCB and (**b**) B-FCB at 28.

An energy-dispersive X-ray (EDX) analysis was performed to observe elemental differences between the FCB and B-FCB specimens. Three elements were the focus of the EDX spectrum tests, namely, Ca, C and O, which are required for production of the CaCO_3_ composite [33]. The results showed that the weight percentage (wt.%) of C and O in the FCB specimens was lower compared to B-FCB. However, the wt.% Ca in FCB was higher than in the B-FCB specimens, as shown in Figure 9a,b. According to these findings, the role of *B. tequilensis* and its enzymes was clearly observed in the comparative changes in the selected elements (wt.%). The results also confirmed that the free atoms of Ca decreased in B-FCB due to the formation of CSH and the deposition of calcium hydroxide (Ca(OH)_2_), which precipitated CaCO_3_. This was due to the combination reaction between Ca(OH)_2_ carbonation and *B. tequilensis* enzymes, which resulted in the acceleration of the CO_2_ sequestration process into the B-FCB pores.

It was also observed that the wt.% of oxygen increased in B-FCB compared to FCB due to the microbial activities, which released oxygen during the precipitation of CaCO_3_. In this study, oxygen was also available in both FCB and B-FCB from to the foaming agent used in the concrete mixture. In a study by Zhang [34], the availability of oxygen in concrete was shown to help the bacteria to precipitate CaCO_3_. Thus, to provide oxygen for microbial CaCO_3_ precipitation inside the bio-concrete, bacteria and oxygen-releasing compounds were compressed together as the core material of the microcapsule.

The elemental concentrations differed depending on the physicochemical conditions [35]. In this study, physical properties such as pore size and distribution differed from one mixture to another, because the density levels were dissimilar. Consequently, the quantity of foaming agent used in each run was different, which affected the concentration of the mineral contents. Additionally, the curing environment, e.g., the CO_2_ concentration and temperature in the chamber, played a significant role in the weight change of the elements in FCB and B-FCB. Hence, it was difficult to compare the elements between different runs. However, a comparison could be more useful between FCB and B-FCB in a similar run with the same physical and curing conditions.

The results of the EDX analyses showed that the composition of the primary elements Ca, O and C confirmed the presence of CaCO_3_. The increase in C and decrease of Ca in B-FCB confirmed the formation of CaCO_3_ on the surface of *B. tequilensis*, which improved its compressive strength [36].

### 4.3. Crystallinity Analysis of FCB and B-FCB

**Figure 10:**
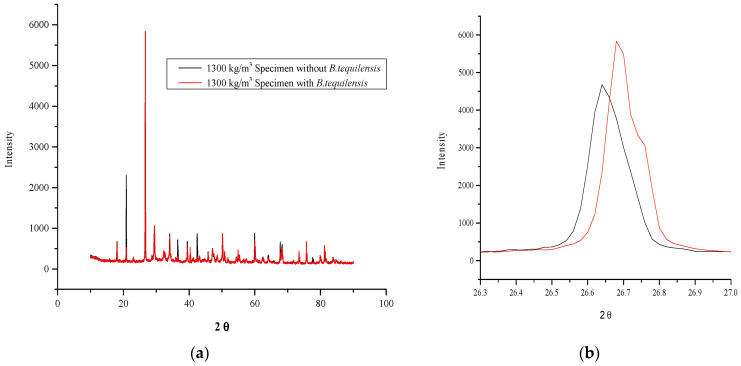

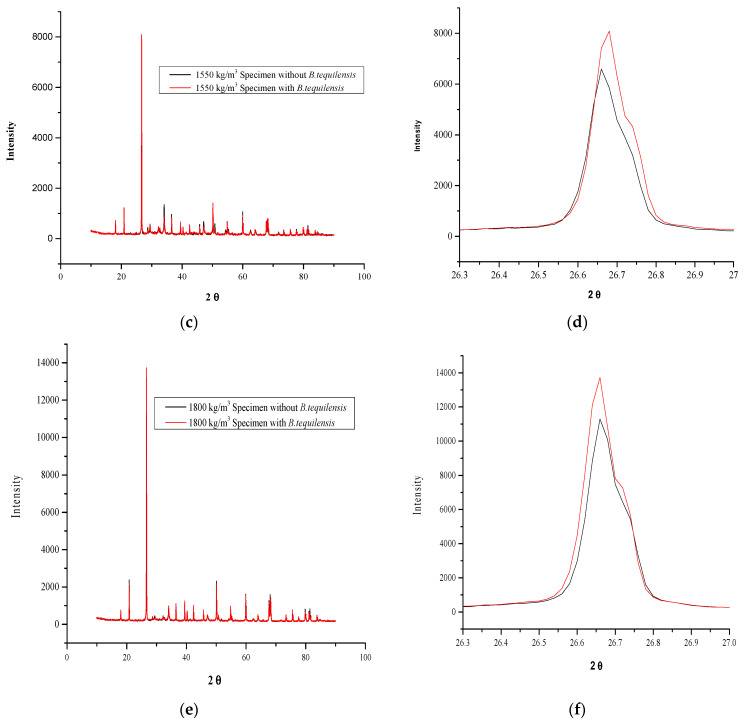
XRD analysis comparison between FCB and B-FCB at 28 days, (**a**) Specimens with 1300 kg/m^3^ of density, (**b**) The highest peak at specimen with 1300 kg/m^3^ of density, (**c**) Specimens with 1550 kg/m^3^ of density, (**d**) The highest peak at specimen with 1550 kg/m^3^ of density (**e**) Specimens with 1800 kg/m^3^ of density, (**f**) The highest peak at specimen with 1800 kg/m^3^ of density.

The XRD technique was used in this study to observe and compare calcite formation in FCB and B-FCB. The results indicated that the D of FCB and B-FCB played the most significant role in the increase or decrease of the XRD intensity. The calcite intensity of the runs at different densities, i.e., 1300 kg/m^3^, 1550 kg/m^3^ and 1800 kg/m^3^, of FCB and B-FCB are demonstrated in Figure 10. The intensity decreased when the density of FCB and B-FCB decreased. Therefore, the highest intensity occurred at 1800 kg/m^3^, and the lowest at 1300 kg/m^3^. At a low density of FCB and B-FCB, air bubbles were generated due to the high reactivity of the foaming agent compared to the high density. As a result, the cement content reduced, resulting in diminished Ca content, which is the key factor in the formation of CaCO_3_. Therefore, the results of the XRD analysis and the figures plotted were classified based on the densities used in this study. Figure 10a,b show that the highest intensity of the FCB specimens was 5500 at 1300 kg/m^3^, while it was 5900 for B-FCB with a similar D, which is lower than the intensity of specimens at 1550 kg/m^3^ and 1800 kg/m^3^. However, the highest intensities for both types of FCB and B-FCB with 1550 kg/m^3^ were 6900 and 8200, as shown in Figure 10c,d, respectively. Figure 10e,f show that at 1800 kg/m^3^, the highest intensity of FCB was almost 11,000, while for B-FCB, it was almost 13,500 with the same level of D.

The results suggest that the crystallinity intensity in B-FCB is higher than that of FCB, which reflects the role of *B. tequilensis* in precipitating CaCO_3_. Moreover, some peaks appeared on the specimens of B-FCB, but were absent in FCB [37], due to the reactions of the *B. tequilensis* enzymes, which integrated the natural sequestration of CO_2_ (resulting in higher CaCO_3_ yield) and the healing process of the specimen pores [37].

## 5. Conclusions

This study presented the use of a statistical design to optimize the compressive strength of B-FCB using *B. tequilensis* as a factor to accelerate CaCO_3_ formation through the reaction of CA and urease enzymes and sequestrated CO_2_. The optimization analysis was carried out to investigate the effects of four key factors (D, CO_2_, B and T) on the compressive strength of B-FCB. Based on the findings of this research, the accuracy of the compressive strength data from 21 runs used in RSM was very high, while the error was below 10%. Factors D, T and CO_2_, as well as the interactions between CO_2_*B and CO_2_*D, had a significant effect on the compressive strength performance of B-FCB. However, D, T and CO_2_*B were the most significant factors affecting the compressive strength results of B-FCB. The highest compressive strength of B-FCB was 8.22 MPa at 28 days, which occurred when CO_2_, B, T and D were at the following levels: 10%, 3 × 10^7^ cell/mL, 27 °C and 1800 kg/m^3^, respectively. In addition, the strong interaction effects between CO_2_*B and compressive strength reflected the relationship between sequestrated CO_2_ and *B. tequilensis* concentration. This, in turn, enhanced the precipitation of CaCO_3_, healed the B-FCB pores, and improved the compressive strength. Therefore, the compressive strength of B-FCB was higher than that of FCB.

## Figures and Tables

**Table 1:** Summary of the chemical composition of the OPC.

Chemical Compound	Concentration (%)
SiO_2_	20.6
Al_2_O_3_	5.4
Fe_2_O_3_	4.2
SO_3_	2.2
K_2_O	0.6
CaO	64.8
MgO	2.2

**Table 2:** *B. tequilensis* concentration as the main factor in (RSM).

Level	Bacteria Concentration (Cell/mL)	*B. tequilensis* Concentration (g/L)	Value Used in (RSM)
Low (−1)	3 × 10^5^	0.001	5
Centre (0)	3 × 10^6^	0.01	6
High (1)	3 × 10^7^	0.1	7

**Table 3:** Design scheme and levels of the process parameters.

Factor Symbol	Parameter	Levels			Unit
		Low (−1)	Centre (0)	High (+1)	
CO_2_	CO_2_ Concentration	10	15	20	(%)
B	*B. tequilensis* concentration	3 × 10^5^	3 × 10^6^	3 × 10^7^	(cell/mL)
T	Temperature	27	33.5	40	(°C)
D	Density of concrete	1300	1550	1800	(kg/m^3^)

**Table 4:** Concrete mixture and RSM design for the optimization experiment.

Run No.	Density (kg/m^3^)	Cement (kg/m^3^)	Fine Sand (kg/m^3^)	Water (L/m^3^)	T (°C)	CO_2_ (%)	B (Cell/mL)
1	1300	553.2	746.8	276.6	27	10	3 × 10^5^
2	1800	766	1034	383	40	10	3 × 10^5^
3	1300	553.2	746.8	276.6	40	20	3 × 10^5^
4	1800	766	1034	383	27	20	3 × 10^5^
5	1300	553.2	746.8	276.6	40	10	3 × 10^7^
6	1800	766	1034	383	27	10	3 × 10^7^
7	1300	553.2	746.8	276.6	27	20	3 × 10^7^
8	1800	766	1034	383	40	20	3 × 10^7^
9	1550	659.5	890.4	329.7	33.5	15	3 × 10^6^
10	1550	659.5	890.4	329.7	33.5	15	3 × 10^6^
11	1550	659.5	890.4	329.7	33.5	15	3 × 10^6^
12	1550	659.5	890.4	329.7	33.5	10	3 × 10^6^
13	1550	659.5	890.4	329.7	33.5	20	3 × 10^6^
14	1550	659.5	890.4	329.7	33.5	15	3 × 10^5^
15	1550	659.5	890.4	329.7	33.5	15	3 × 10^7^
16	1550	659.5	890.4	329.7	27.0	15	3 × 10^6^
17	1550	659.5	890.4	329.7	40.0	15	3 × 10^6^
18	1300	553.2	746.8	276.6	33.5	15	3 × 10^6^
19	1800	766	1034	383	33.5	15	3 × 10^6^
20	1550	659.5	890.4	329.7	33.5	15	3 × 10^6^
21	1550	659.5	890.4	329.7	33.5	15	3 × 10^6^

**Table 5:** Compressive strengths of all samples in the screening stage.

Std. Run No.	Input Variables				
	Uncoded Value				Responses
	Density (kg/m^3^)	Bacteria (Cell/mL)	Temperature (°C)	CO_2_ (%)	Compressive Strength of B-FCB (MPa)
1	1300	3 × 10^5^	27	10	0.90
2	1800	3 × 10^5^	27	20	7.23
3	1800	3 × 10^7^	27	10	8.38
4	1300	3 × 10^7^	27	20	1.02
5	1800	3 × 10^5^	40	10	6.72
6	1300	3 × 10^5^	40	20	0.78
7	1300	3 × 10^7^	40	10	0.61
8	1800	3 × 10^7^	40	20	6.13
9	1550	3 × 10^6^	33.5	15	2.72
10	1550	3 × 10^6^	33.5	15	2.63
11	1550	3 × 10^6^	33.5	15	2.60

**Table 6:** ANOVA analysis of the compressive strength of B-FCB.

Source	DF	Adj SS	Adj MS	*F*-Value	*p*-Value	Effect	Ranking
Model	8	84.964	10.620	2723.220	0.000	-	-
Linear	4	79.991	19.997	5127.640	0.000	-	-
CO_2_	1	0.316	0.316	81.030	0.012	−0.397	5
B	1	0.017	0.017	4.390	0.171	0.092	7
T	1	1.470	1.470	377.080	0.003	−0.857	2
D	1	78.187	78.187	20,048.080	0.000	6.252	1
2-Way Interactions	3	1.062	0.354	90.840	0.011	-	-
CO_2_*B	1	0.546	0.546	140.000	0.007	0.522	3
CO_2_*T	1	0.070	0.070	18.030	0.051	0.187	6
CO_2_*T	1	0.446	0.446	114.490	0.009	−0.472	4
Curvature	1	3.910	3.910	1002.660	0.001	-	-
Error	2	0.007	0.004	-	-	-	-
Total	10	84.972	-	-	-	-	-

**Table 7:** Results of the full design of RSM for compressive strength and carbonation depth.

Std. Run No.	Input Variables				
	Uncoded Value				Responses
	Density (kg/m^3^)	Bacteria (Cell/mL)	Temperature (°C)	CO_2_ (%)	Compressive Strength of B-FCB (MPa)
1	1300	3 × 10^5^	27.0	10	0.9
2	1800	3 × 10^5^	27.0	20	7.23
3	1800	3 × 10^7^	27.0	10	8.38
4	1300	3 × 10^7^	27.0	20	1.02
5	1800	3 × 10^5^	40.0	10	6.72
6	1300	3 × 10^5^	40.0	20	0.78
7	1300	3 × 10^7^	40.0	10	0.61
8	1800	3 × 10^7^	40.0	20	6.13
9	1550	3 × 10^6^	33.5	15	2.72
10	1550	3 × 10^6^	33.5	15	2.63
11	1550	3 × 10^6^	33.5	15	2.60
12	1550	3 × 10^6^	33.5	10	2.55
13	1550	3 × 10^6^	33.5	20	2.56
14	1550	3 × 10^5^	33.5	15	2.16
15	1550	3 × 10^7^	33.5	15	2.31
16	1550	3 × 10^6^	27.0	15	2.54
17	1550	3 × 10^6^	40.0	15	2.12
18	1300	3 × 10^6^	33.5	15	0.81
19	1800	3 × 10^6^	33.5	15	7.2
20	1550	3 × 10^6^	33.5	15	2.54
21	1550	3 × 10^6^	33.5	15	2.59

**Table 8:** ANOVA results of compressive strength (after backward elimination).

Source	Degrees of Freedom	Sum of Squares	Mean Square	*F*-Value	*p*-Value
Model	7	113.265	16.180	413.290	0.000
Linear	4	100.355	25.088	640.820	0.000
CO_2_	1	0.250	0.249	6.380	0.025
B	1	0.027	0.027	0.690	0.421
T	1	1.482	1.482	37.860	0.000
D	1	98.596	98.596	2518.370	0.000
Square	1	11.918	11.917	304.400	0.000
D*D	1	11.918	11.917	304.400	0.000
2-Way Interaction	2	0.993	0.496	12.680	0.001
CO_2_*B	1	0.546	0.546	13.950	0.003
CO_2_*D	1	0.447	0.446	11.400	0.005
Error	13	0.509	0.039	-	-
Lack-of-Fit	10	0.500	0.050	16.570	0.020
Pure Error	3	0.009	0.003		
Total	20	113.774	-	-	-
Standard deviation = 0.197865 R^2^ = 99.55% R^2^ adjusted = 99.31% Predicted R^2^ = 98.06%					

## Data Availability

The results of the study are not placed in any publicly archived datasets.
